# The treatment with non-thermal plasma on HaCaT human keratinocytes can block TNF-ɑ and IFN- ɑ mediated pro-inflammatory gene expressions

**DOI:** 10.1186/2045-7022-5-S1-P14

**Published:** 2015-03-11

**Authors:** Jeong-Hae Choi, Gyoo-Cheon Kim, Jin-Woo Hong

**Affiliations:** 1 Pusan National University, Department of oral anatomy and cell biology, Yangsan; South Korea; 2 Pusan National University, Department of Internal Medicine, Yangsan South Korea

## 

Recently, many possible roles of non-thermal plasma (NTP) has been reported, representing the plasma as a novel tool in the field of dermatology. Unlike other light-based devices, multi-functional elements within the plasma including UVs, ions, reactive oxygen species drives many biological effect of the plasma, such as blood coagulation stimulation, anti-cancer activity and powerful killing effect against several kinds of bacteria, etc. Among these, bactericidal effect of the plasma has been greatly focused because this can help on wound healing, curing from inflammatory skin diseases by minimizing secondary infections. Although many studies dealing with this property of the plasma were continuously evoked, there were no attempts for elucidating the direct effect of the plasma on immune responses. In this study, anti-inflammatory function of NTP was tested by monitoring the chemokine (C-C motif) ligand 17 (CCL17) expressions from HaCaT human keratinocytes. The mere treatment of NTP effectively decreased CCL17 gene expression of normal condition HaCaT cells, and this function was not mediated from the UV exposure. Furthermore, the treatment of NTP also significantly blocked TNF-¥á and IFN-¥ã mediated CCL17, CCL13 and CCL11/eotaxin gene expression. Although the NTP successfully reduced cytokine gene expressions, NTP increased the expression of type-I collagen and VEGF-A genes and dose not induced any kind of damages to the keratinocytes. Taken together, the results of this study represents NTP can safely controls exaggerated immune-reactions by suppressing several gene expressions from keratinocytes activated by pro-inflammatory cytokines, and this novel property of NTP might be helpful for treating several inflammatory skin diseases.

